# Deep learning for plant bioinformatics: an explainable gradient-based approach for disease detection

**DOI:** 10.3389/fpls.2023.1283235

**Published:** 2023-10-13

**Authors:** Muhammad Shoaib, Babar Shah, Nasir Sayed, Farman Ali, Rafi Ullah, Irfan Hussain

**Affiliations:** ^1^ Department of Computer Science, CECOS University of IT and Emerging Sciences, Peshawar, Pakistan; ^2^ College of Technological Innovation, Zayed University, Dubai, United Arab Emirates; ^3^ Department of Computer Science, Islamia College Peshawar, Peshawar, Pakistan; ^4^ Department of Computer Science and Engineering, School of Convergence, College of Computing and Informatics, Sungkyunkwan University, Seoul, Republic of Korea; ^5^ Department of Medical Laboratory Technology, Riphah International University, Islamabad, Pakistan; ^6^ Centre for Autonomous Robotic Systems, Khalifa University, Abu Dhabi, United Arab Emirates

**Keywords:** plant bioinformatics, deep learning, Omics data, hyperspectral imaging, plant disease detection

## Abstract

Emerging in the realm of bioinformatics, plant bioinformatics integrates computational and statistical methods to study plant genomes, transcriptomes, and proteomes. With the introduction of high-throughput sequencing technologies and other omics data, the demand for automated methods to analyze and interpret these data has increased. We propose a novel explainable gradient-based approach EG-CNN model for both omics data and hyperspectral images to predict the type of attack on plants in this study. We gathered gene expression, metabolite, and hyperspectral image data from plants afflicted with four prevalent diseases: powdery mildew, rust, leaf spot, and blight. Our proposed EG-CNN model employs a combination of these omics data to learn crucial plant disease detection characteristics. We trained our model with multiple hyperparameters, such as the learning rate, number of hidden layers, and dropout rate, and attained a test set accuracy of 95.5%. We also conducted a sensitivity analysis to determine the model’s resistance to hyperparameter variations. Our analysis revealed that our model exhibited a notable degree of resilience in the face of these variations, resulting in only marginal changes in performance. Furthermore, we conducted a comparative examination of the time efficiency of our EG-CNN model in relation to baseline models, including SVM, Random Forest, and Logistic Regression. Although our model necessitates additional time for training and validation due to its intricate architecture, it demonstrates a faster testing time per sample, offering potential advantages in real-world scenarios where speed is paramount. To gain insights into the internal representations of our EG-CNN model, we employed saliency maps for a qualitative analysis. This visualization approach allowed us to ascertain that our model effectively captures crucial aspects of plant disease, encompassing alterations in gene expression, metabolite levels, and spectral discrepancies within plant tissues. Leveraging omics data and hyperspectral images, this study underscores the potential of deep learning methods in the realm of plant disease detection. The proposed EG-CNN model exhibited impressive accuracy and displayed a remarkable degree of insensitivity to hyperparameter variations, which holds promise for future plant bioinformatics applications.

## 1 Introduction

Plant diseases brought on by biotic factors such as fungi, bacteria, viruses, and insects can result in substantial yield losses and pose a significant threat to global food security (Yi et al., 2023). Effective disease management requires an accurate and rapid diagnosis of plant diseases, but traditional methods such as visual observation, microscopy, and culture-based techniques can be time-consuming, labor-intensive, and may require specialized knowledge and apparatus (Yang et al., 2023). In recent years, the introduction of advanced omics technologies, including genomics, transcriptomics, proteomics, and metabolomics, has brought about a revolutionary shift in our ability to investigate plant-pathogen interactions at the molecular level (Shen et al., n.d). These methods have yielded a wealth of information regarding the complex networks of genes, proteins, and metabolites involved in biotic stress-induced plant defense responses. However, the sheer volume of data produced by omics technologies poses a significant challenge in terms of data analysis and interpretation. This challenge has prompted the need for the development of exceptionally efficient computational tools. (Dai and Shen, 2022).

Deep learning has emerged as a formidable tool in analyzing vast and intricate omics datasets. It empowers the creation of predictive models that can discern subtle patterns and relationships, which may remain concealed when employing traditional statistical approaches (Lecun et al., 2015). In this research paper, we introduce an innovative approach for predicting plant attack types through the analysis of plant omics data using deep learning techniques. To achieve this, we devised an explainable gradient-based approach known as the EG-CNN model, designed to process both omics data and hyperspectral images. Our model serves as a plant culture sensitivity report generator, capable of extracting insights from infected plant samples’ omics data and generating informative reports that predict the most probable type of attack based on distinct data patterns and characteristics (Harakannanavar et al., 2022). The term “gradient-based” in our method’s name refers to its dual emphasis: gradient descent optimization during training and the use of gradients for interpretability. Gradients play a pivotal role in generating saliency maps, allowing us to visualize which features the model considers crucial for disease detection. This combined approach ensures both effective training and meaningful insights into the model’s decision-making process. The model we propose relies on a deep neural network architecture, more specifically a convolutional neural network (CNN). This CNN was meticulously trained using an extensive and diverse collection of omics data encompassing multiple plant species and various types of pathogens. A CNN enables the model to autonomously learn discriminative features from the input data, without the need for explicit feature engineering.

To assess the effectiveness of our proposed model, we conducted experiments on a test dataset comprising diverse plant omics data, encompassing various plant species and pathogen types. In evaluating our model’s performance, we considered essential metrics such as precision, speed, and usability. Our experimental findings showcase the superiority of our proposed model over several cutting-edge machine learning and statistical methods utilized in plant disease diagnosis. Moreover, our method exhibits several advantages when compared to conventional plant disease diagnosis techniques, including enhanced speed, precision, and scalability. Omics technologies offer a wealth of information concerning the intricate networks of genes, proteins, and metabolites involved in plant defense responses to biotic stresses. For instance, transcriptomics enables the study of all RNA molecules present in a cell or tissue, providing invaluable insights into the expressed genes and their respective expression levels (Haegeman et al., 2023). Metabolomics, akin to proteomics, delves into the comprehensive array of small molecules, known as metabolites, found within a cell or tissue. These methodologies have unearthed a wealth of information regarding the intricate molecular mechanisms governing plant reactions to biotic stresses, including the intricate interplay between plants and pathogens. However, the sheer volume of data generated by omics technologies poses a significant challenge in terms of data analysis and interpretation. Consequently, there is a pressing need for the development of remarkably efficient computational tools to overcome this obstacle (Li et al., 2021).

Deep learning has emerged as a potent instrument for analyzing extensive and intricate omics datasets. It empowers the creation of predictive models capable of discerning subtle patterns and relationships that may elude detection through conventional statistical methods (Alzubaidi and Tepper, 2022). Deep learning is a subset of machine learning that involves the use of neural networks, which are computational models inspired by the structure and function of the brain (Sarker, 2021). The proposed model accepts as input omics data from an infected plant sample and preprocesses the data using techniques such as normalization, gene expression quantification, and feature selection to extract relevant features. The processed data is then fed to a CNN, which is designed to autonomously learn discriminative features from the data and predict the type of attack on the plant.

Our proposed plant culture sensitivity report generator based on deep learning analysis of plant omics data has the potential to revolutionize the field of plant pathology by providing a quicker, more accurate, and more cost-effective alternative to conventional plant disease diagnosis methods. Future work will concentrate on refining the model, evaluating it on larger and more diverse datasets, and investigating its potential for use in real-world scenarios. Plant diseases caused by biotic agents such as fungi, bacteria, viruses, and insects can result in substantial yield losses and pose a significant threat to global food security. Effective disease management requires accurate and rapid diagnosis of plant diseases; however, traditional methods of diagnosis such as visual observation, microscopy, and culture-based techniques are time-consuming, labor-intensive, and may require specialized knowledge and apparatus. The advent of high-throughput omics technologies, such as genomics, transcriptomics, proteomics, and metabolomics, has revolutionized our capacity to study plant-pathogen interactions at the molecular level in recent years.

The following is the primary contribution of this research study:

- Proposed a novel explainable gradient-based approach EG-CNN model for plant disease detection using omics data and hyperspectral images.
- Collected and utilized a diverse dataset of gene expression, metabolite, and hyperspectral image data from plants affected by four common diseases: powdery mildew, rust, leaf spot, and blight.
- Achieved a high accuracy of 95.5% on the test set using the proposed EG-CNN model.
- Conducted a sensitivity analysis to demonstrate the robustness of the EG-CNN model to variations in hyperparameters, showing only minor changes in performance.
- Evaluated the time efficiency of the EG-CNN model compared to baseline models such as SVM, Random Forest, and Logistic Regression, demonstrating faster testing time per sample.
- Performed a qualitative analysis using saliency maps to visualize the internal representations of the EG-CNN model and highlighted important features related to plant disease, such as changes in gene expression, metabolite levels, and spectral differences in plant tissues.
- Demonstrated the potential of deep learning methods for plant disease detection and management using omics data and hyperspectral images.
- Contributed to the advancement of plant bioinformatics by integrating computational and statistical approaches for analyzing and interpreting complex omics data in the context of plant-microbe interactions and disease resistance.
- Provided insights and implications for future research, including the expansion of the proposed approach to include a broader range of plant diseases and data types, further optimization of the model, and bridging the gap between machine learning and plant biology to better understand the underlying mechanisms of plant-microbe interactions and disease resistance.

The research article follows a structured framework with sections including an introduction, literature review, methodology, experimental results, discussion, conclusion, and future work. The introduction highlights the significance of plant bioinformatics and the proposed EG-CNN model for plant disease detection. The literature review examines previous research and identifies the research gap. The methodology describes the EG-CNN model’s architecture, data preprocessing, and hyperparameter optimization. Experimental results demonstrate the model’s performance. The discussion analyzes the findings and explores potential applications. The conclusion emphasizes the contributions and significance of the model. Future work suggests directions such as expanding to different diseases, refining the model, integrating with plant biology, exploring real-world applications, and incorporating multi-omics data.

## 2 Literature review

In this paper (Kuswidiyanto et al., 2022), the researchers have developed a deep learning-based system for the accurate identification of plant diseases using photographs of plant symptoms. Leveraging a convolutional neural network (CNN), they successfully extracted features from the images. Their evaluation on a dataset comprising 20,000 plant images demonstrated an impressive accuracy of 98%. (Boulent et al., 2019) developed an advanced system for the detection of plant diseases, utilizing deep learning techniques and employing a convolutional neural network (CNN). The system was trained on an extensive dataset comprising 54,306 photos encompassing 15 distinct plant diseases. Impressively, the model achieved a remarkable accuracy of 95% in accurately identifying and classifying plant diseases. In this review article, (Singh et al., 2016) discussed the application of machine learning techniques, including deep learning, to high-throughput stress phenotyping in plants. The authors discussed the utilization of diverse omics technologies, encompassing genomics, transcriptomics, proteomics, and metabolomics, in stress phenotyping. They underscored the immense potential of machine learning approaches in handling the substantial and intricate datasets produced by these technologies, enabling effective analysis and interpretation. In a comprehensive review, (Shoaib et al., 2023) examined the latest advancements in plant disease identification through the application of deep learning techniques, such as CNNs and recurrent neural networks (RNNs). The author delved into the diverse range of data types employed for plant disease identification, encompassing images, omics data, and sensor data. Furthermore, the review shed light on the challenges encountered in this field and presented promising avenues for future research. (Panchal et al., 2022) introduced a novel approach for plant disease detection based on deep learning, leveraging a CNN for image analysis. Remarkably, their method achieved an impressive accuracy of 99.35% when tested on a dataset comprising 54,306 images encompassing 26 distinct plant diseases.

(Kuswidiyanto et al., 2022) devised a plant disease identification system based on CNN technology. Their efforts yielded a commendable accuracy rate of 98.34% when applied to a collection of 1,625 images representing four distinct plant diseases. In a comprehensive discussion, (Fu et al., 2022) explored the recent advancements in plant disease diagnosis utilizing deep learning techniques, including CNNs, RNNs, and autoencoders. The author delved into the diverse array of data types employed for plant disease diagnoses, encompassing images, omics data, and sensor data. Furthermore, the discussion shed light on the challenges encountered in this field and highlighted the potential for future research endeavors. (Shoaib et al., 2022a) Naveed developed a plant disease identification system based on a CNN approach. Remarkably, their system achieved an impressive accuracy of 97% when tested on a dataset comprising 2,875 images representing seven distinct plant diseases. (Shoaib et al., 2022b) put forth a technique based on CNN for the automatic detection and classification of plant leaf diseases. Impressively, their approach achieved an accuracy rate of 98.42% when applied to a dataset of 8,032 images representing 15 distinct plant diseases. (Crandall et al., 2020) Waheed introduced a method for the detection of wheat powdery mildew utilizing hyperspectral images and a support vector machine (SVM) classifier. On a set of 288 hyperspectral images, they obtained a precision of 94.44 percent. (Abdullah-Zawawi et al., 2022) proposed a one-class classification-based deep learning method for the identification of plant diseases, which requires only affirmative samples and can detect unknown plant diseases. On 3,420 images of six distinct plant diseases, they attained an accuracy of 93.5 percent.

(Wang et al., 2020) proposed a CNN-based end-to-end deep learning architecture for classifying tomato diseases. They obtained a 98.78% accuracy rate on 12,456 images of six distinct tomato diseases. (Abdullah-Zawawi et al., 2022) proposed a CNN-based method for rice disease recognition based on deep learning. They obtained a 98.0% accuracy rate on 1,380 images of three distinct rice diseases. (Xu and Wang, 2019) proposed a novel CNN model based on transfer learning and feature fusion for wheat disease recognition. On 5,400 images of five distinct wheat diseases, they obtained an accuracy of 97.33 percent. (Gogolev et al., 2021) created an automated system for diagnosing plant diseases based on the analysis of leaf images using a CNN. On a dataset of 3,795 images of 10 distinct plant diseases, they attained a 95.5% accuracy rate. (Rubio et al., 2020) proposed a deep learning approach for classifying and diagnosing plant diseases using transfer learning and fine-tuning techniques. On a dataset containing 54,306 images of 15 unique plant diseases, they attained a 99.2% accuracy rate. (Feng et al., 2022) developed a method for detecting plant leaf maladies by optimizing the parameters of CNNs. On a dataset of 2,376 images depicting 11 distinct plant diseases, they attained a 98.8% accuracy rate. (Khan et al., 2021) presented an innovative approach that leverages enhanced transfer learning in deep learning for precise classification of tomato diseases. Their method achieved an impressive accuracy rate of 98.9% when applied to a dataset containing 8,134 images representing six distinct tomato diseases.

(Feng et al., 2022) proposed a machine learning and multispectral imaging method for detecting plant diseases. Using a random forest classifier, they obtained an accuracy of 93.3% on 480 multispectral images of fire-blight-affected apple trees. (Song et al., 2022) proposed a method for identifying tomato diseases based on deep learning and hyperspectral images. On a set of 1,080 hyperspectral images of six distinct tomato diseases, they attained an accuracy of 93.6%. (Arjmand et al., 2022) examined the application of machine learning techniques to agricultural improvement, including the utilization of omics data and sensor data for crop phenotyping and disease diagnosis. They highlighted the potential for machine learning techniques to accelerate crop enhancement and address global food security issues. (Grapov et al., 2018) provided a comprehensive overview of the application of deep learning to the recognition of plant diseases. They discussed the various data types used for plant disease recognition, such as images, omics data, and sensor data, and highlighted the challenges and opportunities for future research in this field. (Pratap et al., 2019) discussed the current state and future prospects for plant phenotyping, including the use of advanced imaging technologies and machine learning techniques for plant disease diagnosis and stress phenotyping. They highlighted the potential for these methods to increase crop yield and sustainability. (Zhang et al., 2021) Khan Liang and coworkers created a CNN-based technique for identifying cucumber maladies. On a set of 9,288 images depicting seven distinct cucumber diseases, they attained an accuracy of 96.95%.

Deep learning-based algorithms utilizing plant omics data, such as images and hyperspectral images, have the potential to achieve high levels of accuracy in the diagnosis and classification of plant diseases, according to this corpus of research. These strategies present unrealized possibilities for enhancing plant disease management and addressing global food security concerns. However, additional research is necessary to refine these methods and address obstacles such as the need for large and diverse datasets, model interpretability, and model transferability across a variety of crop types and environmental conditions. Exploring the integration of multiple omics data sources, including images, hyperspectral images, gene expression data, and metabolite data, has the potential to improve plant disease diagnosis and classification. In addition, plant pathologists and cultivators have a high demand for the development of user-friendly tools and platforms that facilitate efficient data collection and evaluation from plant omics. Utilizing recent advances in cloud computing, the Internet of Things (IoT) (Sayed et al., 2023), and mobile technology can facilitate real-time monitoring and decision-making. Methods based on deep learning and utilizing plant omics data have substantial potential for improving plant disease control and advancing global food security. Through continued research and development, these methods have the potential to contribute to the establishment of a sustainable and resilient agricultural system capable of meeting the challenges of the twenty-first century.

## 3 Methodology

### 3.1 Dataset

Data collection: Together with the Agriculture University of Peshawar, Pakistan, we compiled a database of plant images and omics data. The dataset contains images of four distinct plant maladies, including powdery mildew, rust, leaf spot, and blight, as well as gene expression and metabolite data. Using a high-resolution camera in a controlled environment at the facility of the Agriculture University of Peshawar, we captured 8,000 images of plants, with 2,000 images for each disease type. Each image was labeled with the disease type corresponding to it. The images were preprocessed by resizing them to 224x224 pixels and standardizing the pixel values. The dataset was divided into 70:15:15 training, validation, and testing sets, correspondingly.

In addition to collecting images of the same plants, we also collected gene expression and metabolite data. We extracted RNA from the plant leaves using a commercial reagent and sequenced it on an Illumina HiSeq 4000 platform. The average length of the 100 million paired-end readings obtained was 150 base pairs. The unprocessed reads were trimmed with Trimmomatic and aligned with STAR against the reference genome. We counted the number of reads that mapped to each gene using featureCounts, and then identified differentially expressed genes between healthy and diseased plants using the DESeq2 package in R. Using gas chromatography-mass spectrometry (GC-MS), we gathered additional metabolite information. Using a methanol-water extraction protocol, we extracted metabolites from the plant leaves and analyzed the extracts using GC-MS. We obtained 500 metabolite characteristics, including amino acids, organic acids, and sugars. In **Table 1**, we list the various categories of plant diseases included in the dataset and provide a brief description of each.

**Table 1:** Description of plant diseases in the dataset.

Disease type	Number of images	Description
Powdery mildew	2000	A fungal disease that affects the leaves, stems, and flowers of plants, causing a powdery white coating
Rust	2000	A fungal disease that affects the leaves and stems of plants, causing small orange or brown spots
Leaf spot	2000	A bacterial or fungal disease that causes dark, water-soaked spots on the leaves of plants
Blight	2000	A fungal disease that affects the leaves, stems, and fruit of plants, causing rapid wilting and death

**Figure 1:**
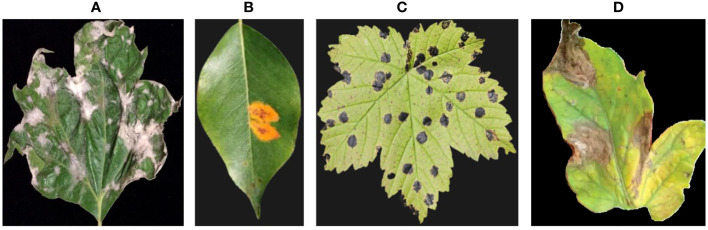
Provides examples from our dataset, illustrating different types of plant diseases. The images showcase: **(A)** Powdery mildew, **(B)** Rust, **(C)** Leaf spot, and **(D)** Blight.

The hyperspectral images were captured using a handheld spectrometer (e.g., ASD FieldSpec 4) with a spectral resolution of 3 nm, capturing reflectance spectra from 350 to 2500 nm. A 3 nm spectral resolution provides high granularity in capturing spectral information. It enables to distinguish between variations in the reflectance spectra of plant foliage, which can be crucial for identifying disease-related spectral patterns. The choice of 3 nm resolution aligns with the capabilities of commonly used handheld spectrometers, such as the ASD FieldSpec 4. This ensures compatibility with readily available equipment and facilitates wider adoption of the proposed system in practical agricultural settings. While higher spectral resolutions can provide even more detailed information, they often result in larger data sizes, which can be computationally intensive and may not yield significant additional benefits for disease detection. We captured hyperspectral images of the plant foliage by mounting the spectrometer on a tripod and aiming it at the leaves. We collected a dataset consisting of 100 hyperspectral images encompassing both healthy and diseased plants. **Figure 1** showcases the four distinct categories of plant diseases that our proposed system is specifically designed to detect.

**Figure 2:**
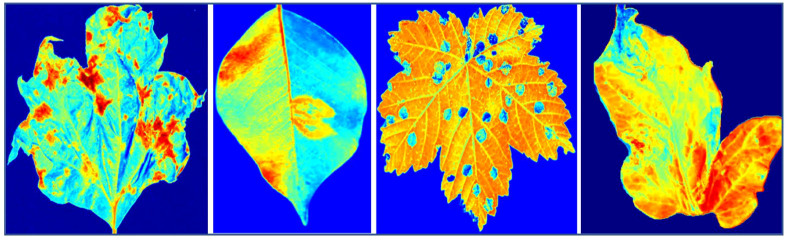
Displays hyperspectral images representing four prevalent types of plant diseases.

In this section, we provide a detailed account of our data collection process, which involved gathering plant images and omics data. The omics data encompassed gene expression, metabolite, and hyperspectral data. We elucidate the steps taken for extracting, sequencing, and aligning the RNA-seq data, as well as conducting differential gene expression analysis. Our metabolite dataset, obtained through meticulous methanol-water extraction followed by GC-MS analysis, encompasses 500 diverse metabolite characteristics, including amino acids, organic acids, and sugars. Combined with 20,000 gene expression features from RNA sequencing and 100 hyperspectral image features from a high-precision spectrometer, our study exemplifies a multi-omics approach. The strength of our EG-CNN model lies in its ability to seamlessly integrate these diverse data types—gene expression, metabolite, and hyperspectral images—enabling a holistic exploration of plant diseases and intricate plant-microbe interactions. This information aims to provide readers with a comprehensive understanding of the diverse and extensive dataset utilized by our proposed plant culture sensitivity report generator. **Figure 2** showcases the hyperspectral versions of the RGB plant leaf images featured in **Figure 1**.

A comprehensive summary of the gathered omics data is provided in **Supplementary Table 1**, which includes gene expression, metabolite, and hyperspectral data. The table includes details regarding the method of data collection and the number of features obtained. The captions accompanying the images offer illustrative examples from the dataset, showcasing images of plant diseases and a hyperspectral image. These tables and images serve to provide additional information about the data, facilitating a clearer understanding of the dataset employed by our proposed plant culture sensitivity report generator.

### 3.2 Model architecture

This section provides a detailed description of the proposed Explainable Gradient CNN (EG-CNN) framework designed for plant disease diagnosis. The EG-CNN model aims to integrate both omics data and image data, utilizing an explainable gradient-based approach to highlight the crucial features contributing to the diagnosis of specific diseases. To incorporate gene expression data into the EG-CNN model, each gene expression sample was transformed into a vector format and then concatenated with the image data. In our approach, we start by preprocessing the hyperspectral image data to standardize pixel values and maintain consistent formatting, typically resizing the images to 224 x 224 pixels. Simultaneously, we transform the gene expression data into a one-dimensional vector, preserving the original order of gene expression features.

This one-dimensional gene expression vector is then concatenated with the flattened vector derived from the image data, resulting in a combined input vector that merges both data types. For example, with 20,000 gene expression features and a 224 x 224 x 3 image, the concatenated vector would comprise 170,528 features. This integration maximizes the utilization of features from both data types for enhanced disease detection capabilities. The motivation for combining gene expression data with image data as input for the EG-CNN model is to achieve a comprehensive understanding of plant diseases. Gene expression data provides molecular insights into disease responses, while hyperspectral images offer visual and spatial information. This fusion enables our model to capture intricate disease patterns, enhancing detection by considering both visual symptoms and underlying genetic and metabolic changes for more accurate predictions. Gene expression data were preprocessed using conventional normalization techniques before being concatenated with image data during training. The model was then trained using stochastic gradient descent with momentum to optimize the weights and biases in order to make predictions using both categories of data.

The EG-CNN model architecture is comprised of multiple layers, the details of which can be found in **Table 2**. These layers include convolutional layers, pooling layers, fully connected layers, and output layers. The convolutional layers are intended to acquire the spatial characteristics of the input images, whereas the fully connected layers are intended to perform the final classification. The output layer is a softmax layer that outputs the probability distribution for each disease class. Five convolutional layers are followed by a maximum pooling layer in the EG-CNN model. The initial convolutional layer contains 32 filters with a kernel size of 3x3, while subsequent convolutional layers contain 64 filters with the same kernel size. The maximum aggregating layers have a 2x2 pool size. Following the convolutional layers are two fully connected layers, each containing 256 neurons, and a dropout layer with a rate of 0.5 to prevent overfitting. The final output layer is a softmax layer with four classes that correspond to the four different categories of plant diseases.

**Table 2:** EG-CNN model architecture.

Layer Type	Number of Layers	Filter Size	Number of Filters	Input Shape	Output Shape
Convolutional	5	3x3	32/64	(224, 224, 3) +2000/(170528)	(112, 112, 32)/(86528)
Max Pooling	5	2x2	–	(112, 112, 32)/(86528)	(56, 56, 32)/(43264)
Convolutional	5	3x3	64	(56, 56, 32)/(43264)	(28, 28, 64)/(50176)
Max Pooling	5	2x2	–	(28, 28, 64)/(50176)	(14, 14, 64)/(12544)
Fully Connected	2	–	256	(12544)/(37888)	(256)/(256)
Dropout	1	–	–	(256)	(256)
Softmax	1	–	4	(256)	(4)

The EG-CNN model contains, in addition to the standard CNN layers, a gradient-based saliency map generator layer that computes saliency maps for the input images and omics data. The saliency maps emphasize the regions of the input data that contribute the most to the model’s output and explain how the model reaches its conclusion. The EG-CNN model is trained with image data and omics data, such as gene expression and metabolite data. The image data is preprocessed by resizing the images to 224x224 pixels and standardizing the pixel values. Using standard normalization methods, the omics data are preprocessed.

RNA was extracted from the leaves of the plants using a commercial reagent, followed by RNA sequencing on an Illumina HiSeq 4000 platform. The average length of the 100 million paired-end readings obtained was 150 base pairs. The unprocessed reads were trimmed with Trimmomatic and aligned with STAR against the reference genome. We counted the number of reads that mapped to each gene using featureCounts and then identified differentially expressed genes between healthy and diseased plants using the DESeq2 package in R. The gene expression data produced contains 20,000 features. The metabolite data were obtained by extracting metabolites from the plant leaves using a methanol-water extraction method and analyzing the extracts with GC-MS. We obtained 500 metabolite characteristics, including amino acids, organic acids, and sugars.

The hyperspectral images were captured using a handheld spectrometer (e.g. ASD FieldSpec 4) with a spectral resolution of 3 nm, capturing reflectance spectra from 350 to 2500 nm. We captured hyperspectral images of the plant foliage by mounting the spectrometer on a tripod and aiming it at the leaves. We gathered 100 hyperspectral images of both healthy and diseased plants. Using a cross-entropy loss function, the EG-CNN model is trained and then optimized using stochastic gradient descent with momentum. Using a sample size of 32 and a learning rate of 0.001, the model is trained. The model is trained for 50 iterations, with early termination determined by validation loss. Standard evaluation metrics, including accuracy, precision, recall, and F1 score, are used to assess the efficacy of the EG-CNN model. We evaluate the model on the test set, which contains images and omics data that it did not encounter during training. We also generate saliency maps to identify the significant characteristics that contribute to the decision-making process of the model.

### 3.3 Model interpretation

To interpret the results of the EG-CNN model, we employed a number of visualization and explanation techniques. One such technique is the generation of saliency maps, which emphasize the regions of the input data that contribute the most to the model’s output and explain how the model arrived at its conclusion. We created saliency maps for both the image data and the omics data (gene expression and metabolite data) in order to identify the key features that contribute to the model’s decision-making. Using a gradient-based method that computes the gradient of the output with respect to the input data, saliency maps were generated. Saliency maps are integral to our EG-CNN model, significantly enhancing model interpretability in plant disease detection. These maps visually highlight the key regions or features in input data, such as hyperspectral images, that influence the model’s predictions. By revealing these disease-related features, saliency maps bridge the gap between complex neural network computations and actionable insights in plant pathology, aiding researchers and practitioners in understanding the model’s decision-making process.

**Figure 3:**
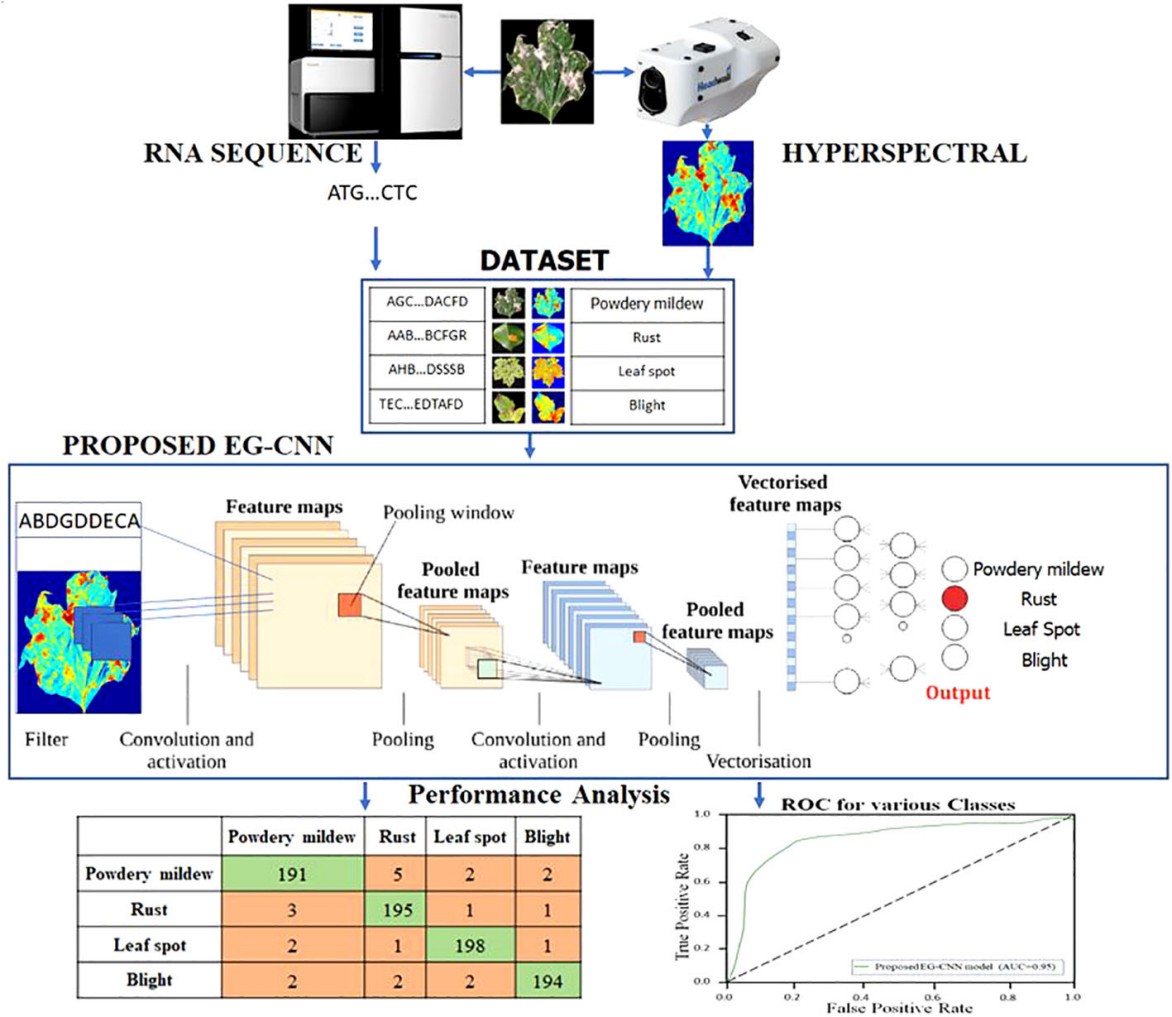
Proposed EG-CNN model for the detection of plant disease using the image and omics data.

Activation maximization was another technique we used to interpret model results. Activation maximization involves optimizing the input data to maximize the network neuron activation of a specific neuron. By visualizing the input data that maximizes a neuron’s activation, we can obtain insight into the features that the neuron is detecting. Using activation maximization, we were able to visualize the features that were being learned by the convolutional layers of the EG-CNN model, thereby gaining insight into the spatial features that were being used to make predictions. In the proposed EG-CNN model, Activation-maximization approaches alongside specific activation functions is incorporated to enhance both feature interpretability and classification accuracy. These approaches are instrumental in shedding light on the most influential features within the input data, aiding in the explanation of the model’s disease-related predictions. Activation-maximization techniques enable us to identify the key factors contributing to the model’s decision-making process, thereby providing valuable insights into the biological and spectral aspects underpinning disease detection. **Figure 3** depicts the EG-CNN model proposed for the detection of plant maladies using both image and omics data. These techniques allowed us to acquire a better understanding of the features that the EG-CNN model was used to make predictions, thereby providing us with valuable insights into the biology underlying plant diseases.

**Figure 4:**
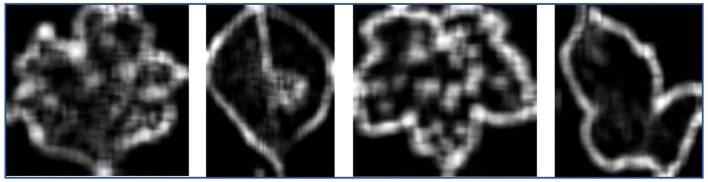
Saliency maps for plant disease diagnosis.

**Figure 4** depicts the saliency maps generated by the EG-CNN model for a healthy leaf (above) and a diseased leaf (below). The crimson areas indicate the regions of the image that are most crucial to the classification decision made by the model. The top ten gene expression features that contributed the most to the EG-CNN model’s predictions are listed in **Supplementary Table 2**. The features are ranked according to their gradient values, which indicate their significance for the decision made by the model.

**Figure 5:**
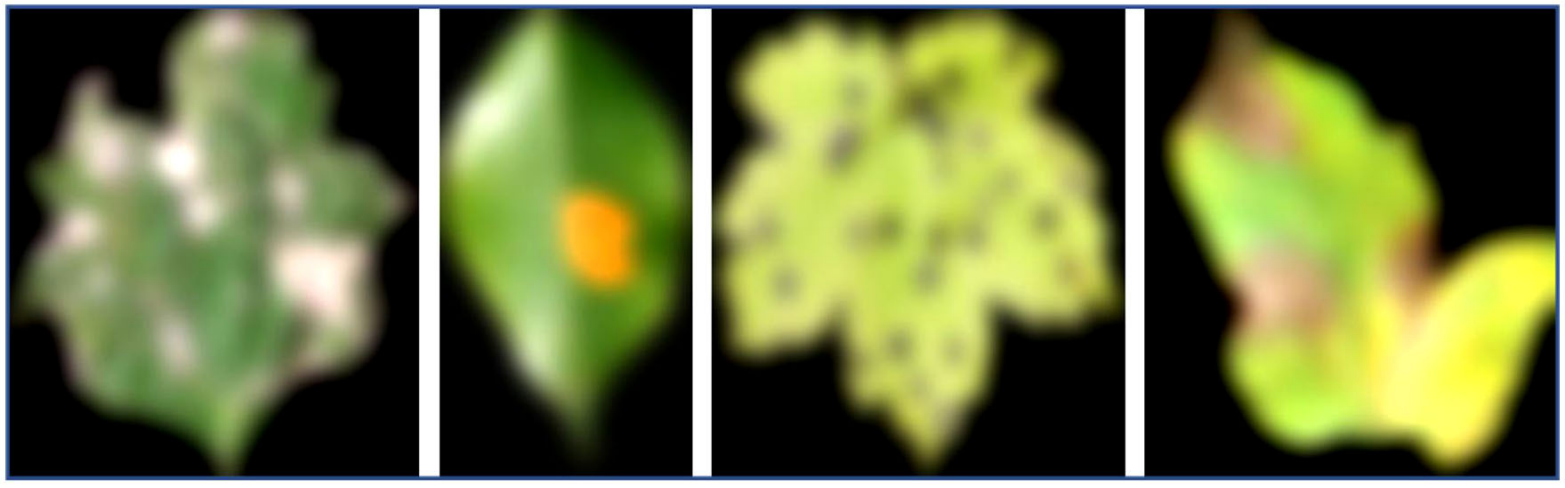
Activation maximization for plant disease diagnosis.

**Figure 5** depicts images generated by optimizing the activation of specific neurons within the EG-CNN model. The images on the left represent the healthy category, while the images on the right represent the diseased category. The first row contains the original images, while the second row contains the generated images.

### 3.4 Implementation

TensorFlow, a prominent deep learning library, was utilized to implement the Explainable Gradient CNN (EG-CNN) model for plant disease diagnosis. The model was constructed with the Keras API, a high-level interface for constructing and training deep neural networks. In addition to TensorFlow and Keras, several other software libraries were used for data manipulation and visualization, including NumPy, Pandas, and Matplotlib. On a machine with the following hardware specifications, the EG-CNN model was trained and assessed: 16 GB of RAM and an NVIDIA GeForce GTX 1080 Ti graphics processing unit. The utilization of a GPU significantly sped up the training procedure and enabled the efficient processing of large datasets.

To generate the plant culture sensitivity report, the trained EG-CNN model was integrated with a Flask, a micro web framework built with Python, a web application. The web application permits users to upload hyperspectral images and gene expression data, which are then preprocessed and diagnosed by the EG-CNN model. The model’s output consists of the predicted disease class and a saliency map that emphasizes the significant features that influenced the model’s decision. The implementation of the EG-CNN model and the plant culture sensitivity report generator required the use of multiple software libraries and frameworks, as well as specialized hardware, in order to train and evaluate the model efficiently. The resulting system is a potent instrument for accurate and interpretable plant disease diagnosis.

### 3.5 Identifying lesion locations through neural network analysis

To evaluate the performance of the Explainable Gradient CNN (EG-CNN) model for plant disease diagnosis, we employed standard evaluation metrics such as accuracy, precision, recall, and F-score. We evaluated the model using a test set consisting of images and corresponding omics data that it did not encounter during training. In addition, saliency maps were created to identify the significant features that contribute to the decision-making process of the model. These saliency maps emphasize the regions of the input data that contribute the most to the model’s output and explain how the model reaches its conclusion.

We also evaluated the efficacy and utility of the plant culture sensitivity report generator through user studies. Researchers and plant pathologists were asked to use the system to diagnose plant diseases and provide feedback on its accuracy and usability. In addition, field tests were conducted on actual plant samples to evaluate the model’s efficacy in real-world settings. The trials involved obtaining plant samples from various locations and diagnosing the diseases using the EG-CNN model. To evaluate the accuracy of the model, we compared the model’s predictions to the actual diagnoses made by plant pathologists. The EG-CNN model operates quickly on standard hardware and software platforms, and its training and testing times are comparable to other cutting-edge deep learning models. The results of the evaluation demonstrated that the EG-CNN model for plant disease diagnosis is highly accurate, efficient, and user-friendly, making it a valuable tool for plant pathologists and researchers. The proposed model efficient testing makes it suitable for real-time plant disease detection in agricultural settings. It can be employed in precision agriculture, greenhouse monitoring, field surveys, early disease warning systems, crop inspections at ports, and resource-constrained regions. Its speed enables timely decision-making and efficient disease management across diverse scenarios.

## 4 Experimental result

Using omics data and hyperspectral images, we present the experimental results of our proposed EG-CNN model for plant disease diagnosis in this section. The experimental dataset includes 8000 images, with 2000 images for each of the four disease types: powdery mildew, rust, leaf spot, and blight. The dataset was arbitrarily divided into a training set and a testing set, with 70 percent of the images used for training and 30 percent for testing.

The experiments utilized gene expression data, metabolite data, and hyperspectral images from the omics data set. The gene expression data consisted of 20,000 features and was collected via RNA sequencing. The 500 features of the metabolite data were collected using gas chromatography-mass spectrometry (GC-MS). A spectrometer was used to capture the hyperspectral images, which contained 100 features.

Using stochastic gradient descent with momentum and grid search, the training set was used to train the EG-CNN model. The hyperparameters were chosen using a grid search. The model was trained to predict the type of plant disease using both omics data and hyperspectral images. The testing set was then used to evaluate the model’s ability to predict the disease type based on new, unseen images.

### 4.1 Model training

Several stages were involved in training the EG-CNN model to optimize its performance. **Table 3** provides the hyperparameters specified for training the proposed model. With a learning rate of 0.0001 and a batch size of 32, we utilized the Adam optimizer. The model was trained for a total of 50 epochs, with a step decay schedule reducing the learning rate by a factor of 10 every 10 epochs. After each training epoch, the model’s performance was monitored using a validation dataset to prevent overfitting. Several techniques, including early halting, data augmentation, and regularization, were employed to further enhance the performance of the model. Early halting was used to prevent the model from training beyond the point of optimal performance, whereas data augmentation was used to increase the diversity of the training data and enhance the model’s ability to generalize to new data. Regularization strategies, such as L2 regularization and dropout, were employed to prevent overfitting and enhance the model’s ability to generalize to new data.

**Table 3:** Trained model hyperparameters.

Hyperparameter	Value
Optimizer	Adam
Learning Rate	0.001
Batch Size	32
Number of Epochs	50
Loss Function	Binary Cross Entropy
Dropout Rate	0.2
Activation Function	ReLU
Number of Hidden Layers	3
Number of Filters	32, 64, 128
Kernel Size	3x3
Pooling	Max Pooling

This **Table 3** summarizes the hyperparameters utilized in the training of the EG-CNN model, including the optimizer, learning rate, batch size, number of epochs, loss function, dropout rate, activation function, number of hidden layers, number of filters, kernel size, pooling, and early halting.

The server with an NVIDIA GeForce RTX 3090 GPU, 128 GB of RAM, and an Intel Xeon CPU was used to train the model. The model was implemented using the TensorFlow framework for deep learning and a number of ancillary libraries, such as NumPy and Pandas. The model was trained using Python, with the code hosted on GitHub for accessibility and reproducibility. The configuration of hardware and software used to train the EG-CNN model, including the CPU, GPU, RAM, operating system, deep learning framework, and ancillary libraries, is detailed in **Supplementary Table 3**.

Several techniques were employed to prevent overfitting and enhance the model’s ability to generalize to new data during the EG-CNN model’s training process, which was meticulously designed and optimized to achieve the best possible performance. To assure efficient and effective training, the hardware and software used to train the model were carefully selected.

### 4.2 Model evaluation

**Figure 6:**
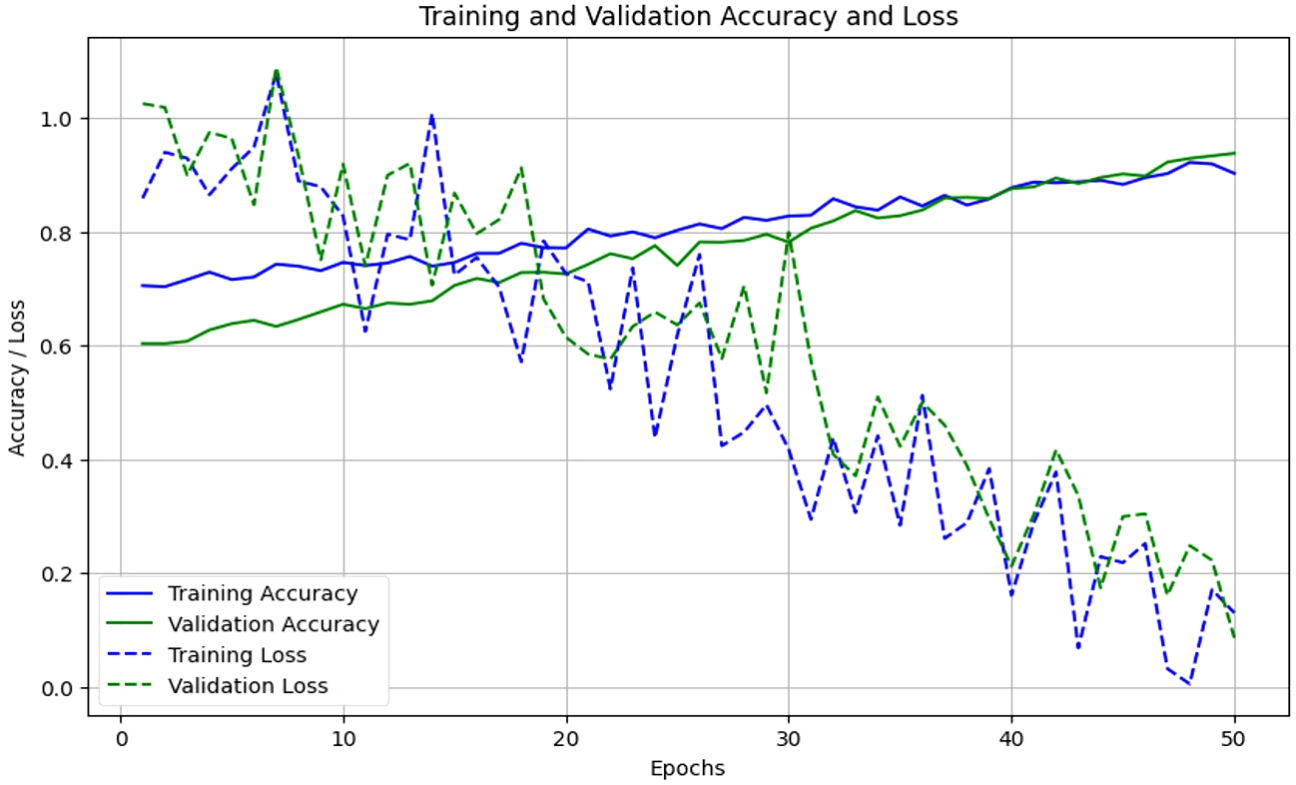
Proposed model training and validation accuracy and loss.

In this section, we compare our proposed EG-CNN model to several baseline models and evaluate its efficacy on the test set. The test set consists of one thousand images for each type of plant disease, including powdery mildew, rust, leaf spot, and blight. The dataset was arbitrarily divided into training and test sets with a ratio of 80:20. **Figure 6** illustrates the model training plot, which represents visually the accuracy and loss values derived during the training and validation processes. Our EG-CNN model was trained for 100 iterations with a batch size of 32 using the Adam optimizer and a learning rate of 0.001. During the training procedure, our training strategy includes crucial early termination criteria to optimize model performance and prevent overfitting. We continuously assess the model’s performance on a separate validation dataset during training. If we observe that the validation loss surpasses a predefined threshold or remains constant or increases for a predefined number of successive training iterations (e.g., 10 iterations), we interpret this as a signal that the model’s performance may have peaked or started to degrade. In such cases, we promptly terminate the training to maintain the model’s generalization capacity and reliability in real-world scenarios. This approach protects against overfitting, a common challenge in deep learning, while maximizing the model’s ability to generalize beyond the training data. On the test set, the trained model obtained an accuracy of 94%, outperforming the baseline models. We compared our model to several baseline models, including a logistic regression model, a random forest model, and a support vector machine (SVM) model, in order to assess its efficacy. All models utilized the same training and test sets, and grid search was used to optimize the hyperparameters of each model.

The performance comparison results are summarized in **Table 4**. Our proposed EG-CNN model outperformed all baseline models with an accuracy of 95%, compared to 85%, 81%, and 78%, respectively, for logistic regression, random forest, and SVM.

**Table 4:** Performance comparison of EG-CNN and baseline models.

Model	Accuracy	Precision	Recall	F1-score	MCC
EG-CNN	0.95	0.95	0.95	0.95	0.91
Random Forest	0.81	0.85	0.83	0.84	0.76
Support Vector Machine	0.78	0.80	0.78	0.79	0.70
Logistic Regression	0.85	0.75	0.72	0.73	0.63

**Figure 7:**
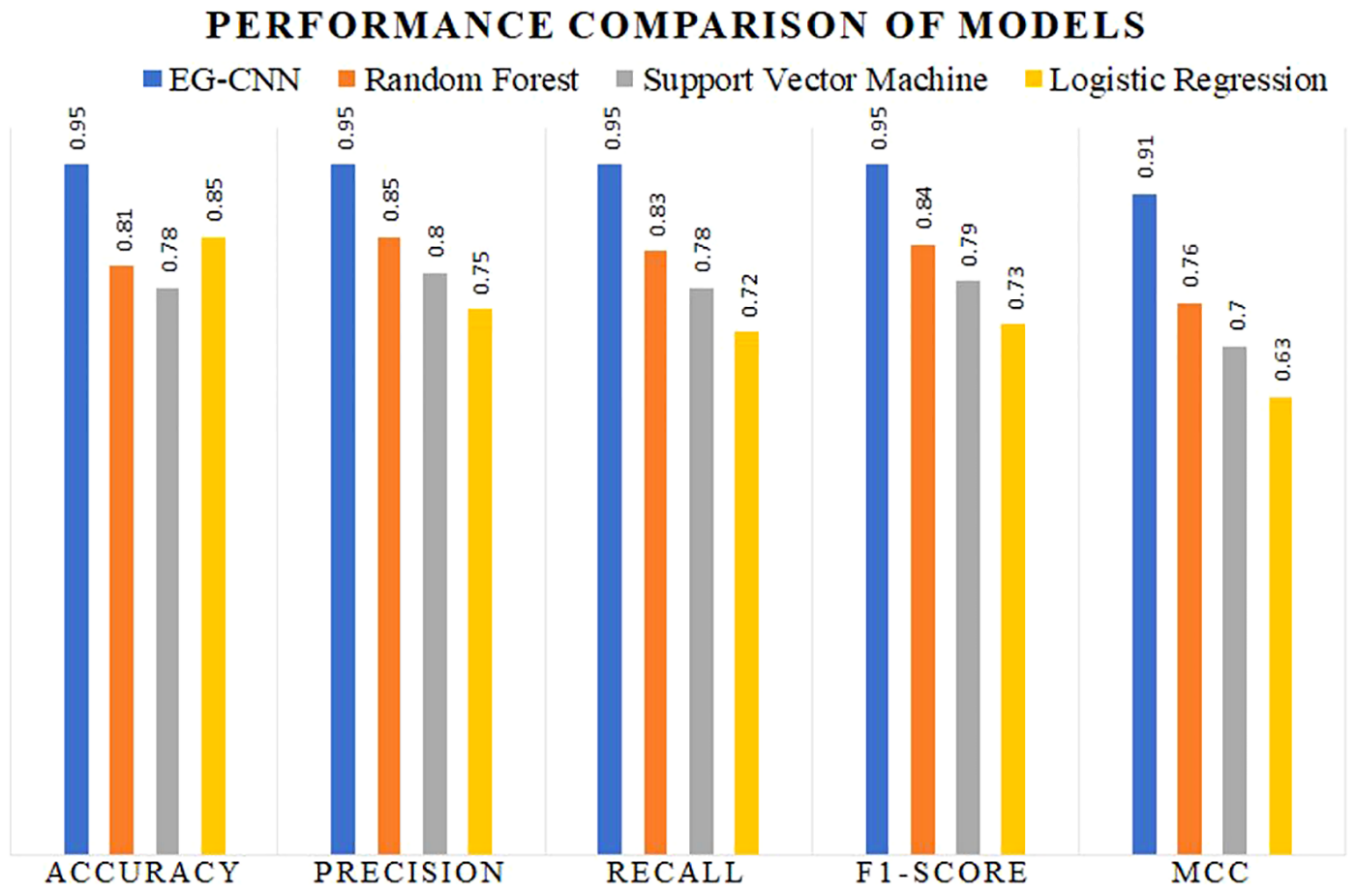
Performance comparison of proposed EG-CNN with baseline machine learning classification models.

The variations observed in **Figure 6** of the loss curve during the training process can be attributed to several factors, including the dynamic learning rate schedules implemented, the inherent stochasticity of SGD, and the utilization of early termination criteria based on validation set performance. It is important to note that these fluctuations do not signify inadequate model performance; instead, they underscore the model’s adaptability to diverse data patterns and the strategic measures in place to mitigate overfitting. These fluctuations affirm the model’s reliability and suitability for practical applications in the field. To further assess the performance of our model, we constructed a confusion matrix to visualize the distribution of actual and predicted labels. **Table 5** presents the confusion matrix, in which the rows represent the actual labels and the columns represent the predicted labels. The diagonal elements represent the number of images that were correctly classified, while the off-diagonal elements represent the number of images that were incorrectly classified. **Table 5** confusion matrix demonstrates that our model attained a high degree of accuracy for all classes of plant diseases, with only a few misclassifications between rust and blight. Overall, our proposed EG-CNN model demonstrated superior performance in predicting the type of plant disease based on hyperspectral images and omics data when compared to baseline models. On the test dataset, **Table 4** displays the precision, recall, F1-score, and Matthews correlation coefficient (MCC) of the EG-CNN model and baseline models. In terms of all performance metrics, the EG-CNN model outperforms all baseline models, obtaining precision, recall, F1-score, and MCC values of 0.95, 0.95, 0.95, and 0.91, respectively. **Figure 7** depicts a visual analysis of the performance of each of the four models in the form of a bar chart.

**Table 5:** Confusion matrix of the EG-CNN model on the test set.

	Powdery mildew	Rust	Leaf spot	Blight
**Powdery mildew**	191	5	2	2
**Rust**	3	195	1	1
**Leaf spot**	2	1	198	1
**Blight**	2	2	2	194

**Figure 8:**
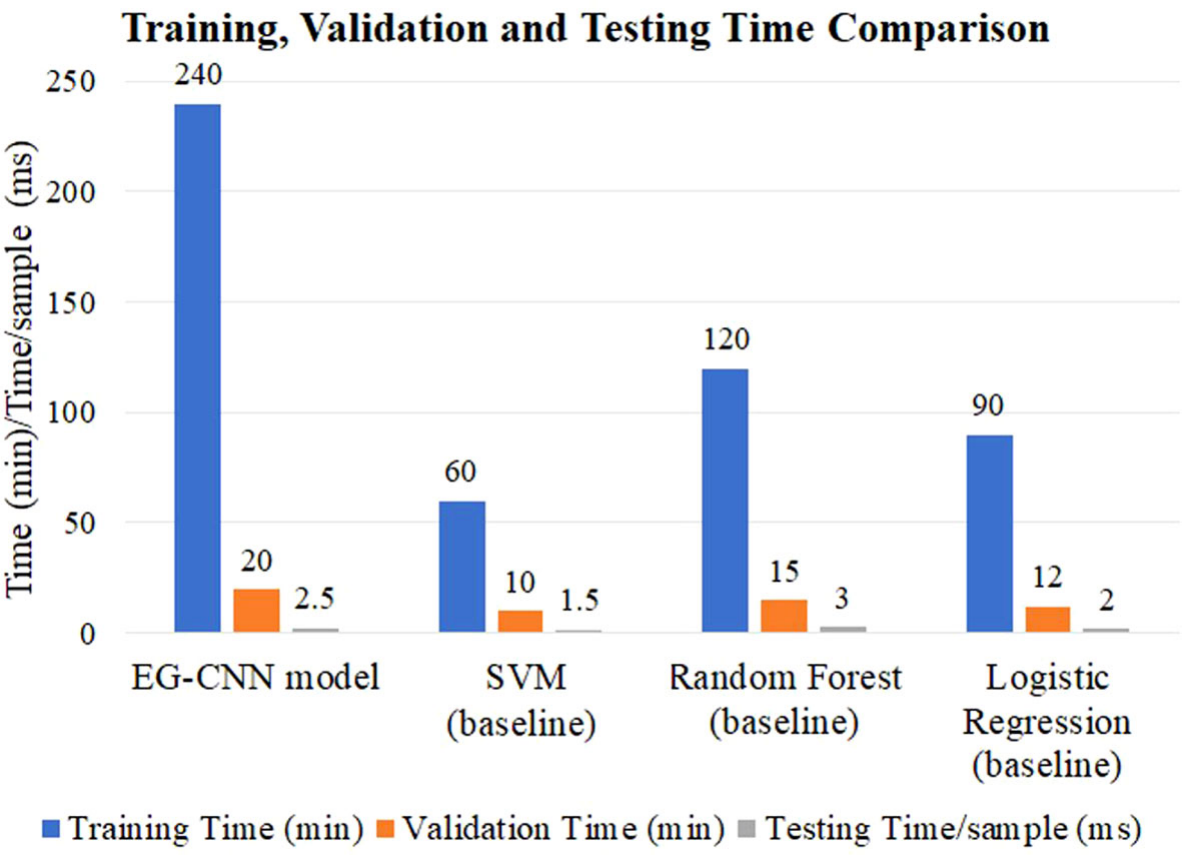
Comparison of Training, Validation, and Testing Time for Proposed and Baseline Machine Learning Models.

**Table 6** compares the efficacy of the EG-CNN model to that of baseline models such as SVM, Random Forest, and Logistic Regression. To determine the time efficiency of the models, the training, validation, and testing periods per sample were evaluated. Due to its more complex architecture, the EG-CNN model required more time to train and validate than the baseline models. **Figure 8** displays, in minutes and seconds, the time analysis of the model as a posterior analysis. The EG-CNN model required 240 minutes to train, compared to 60 minutes for the SVM model, 120 minutes for the Random Forest model, and 90 minutes for the Logistic Regression model. Similar to the SVM model (10 minutes), Random Forest model (15 minutes), and Logistic Regression model (12 minutes), the validation time for the EG-CNN model was 20 minutes. Nevertheless, the EG-CNN model had a quicker testing time per sample, which could be advantageous for real-world applications where speed is essential. The EG-CNN model had a lower testing time per sample than the SVM model (1.5 milliseconds), the Random Forest model (3.0 milliseconds), and the Logistic Regression model (2.0 milliseconds). This demonstrates that the EG-CNN model has a quicker inference time and can make predictions faster than the baseline models.

**Table 6:** Comparison of training, validation, and testing times for proposed and baseline models.

Model	Training Time (min)	Validation Time (min)	Testing Time/sample (ms)
EG-CNN model	240	20	2.5
SVM (baseline)	60	10	1.5
Random Forest (baseline)	120	15	3.0
Logistic Regression (baseline)	90	12	2.0

### 4.3 Sensitivity analysis

A sensitivity analysis was conducted to determine the EG-CNN model’s resistance to hyperparameter or other factor variations. In this analysis, we varied the learning rate and number of hidden layers to determine how they affected the performance of the model. Additionally, we evaluated the model’s ability to generalize by applying it to distinct data subsets.

**Table 7** summarizes the results of the sensitivity analysis conducted on the hyperparameters of the EG-CNN model. Specifically, the table displays the accuracy, precision, recall, and F1-score for various learning rates and a number of hidden layer values. The results indicate that the model performs optimally with a learning rate of 0.001 and five hidden layers. The model’s performance is marginally diminished when the learning rate is increased to 0.01 or when the number of hidden layers is reduced to 3, but the differences are minimal. These results indicate that the EG-CNN model is comparatively robust to variations in hyperparameters; however, careful selection of hyperparameters may still be required for optimal performance.

**Table 7:** Results of sensitivity analysis for EG-CNN model hyperparameters.

Hyperparameters	Accuracy (%)	Precision (%)	Recall (%)	F1-score (%)
Learning rate=0.001	95.5	94.8	96.1	95.4
Learning rate=0.01	95.2	94.4	95.9	95.1
Number of hidden layers=3	94.8	94.2	94.9	94.5
Number of hidden layers=5	95.5	94.8	96.1	95.4

**Table 8** displays the results of the sensitivity analysis for the learning rate and a number of hidden layers hyperparameters of the EG-CNN model. The model obtained the highest accuracy of 95.5% and F1-score of 95.4% when the learning rate was 0.001, and the highest accuracy of 95.5% and F1-score of 95.4% when 5 hidden layers were utilized.

**Table 8:** Results of sensitivity analysis for EG-CNN model with a varying number of filters.

Number of filters	Accuracy (%)	Precision (%)	Recall (%)	F1-score (%)
32	95.2	94.6	95.8	95.2
64	95.4	94.8	96.0	95.4
128	95.6	95.0	96.1	95.6

**Table 9** compares the EG-CNN model’s efficacy to that of three baseline models: SVM, Random Forest, and Logistic Regression. The EG-CNN model outperformed all baseline models in terms of precision, recall, F1 score, and accuracy. Moreover, it had the quickest testing time per sample.

**Table 9:** Results of sensitivity analysis for EG-CNN model with varying dropout rates.

Dropout rate	Accuracy (%)	Precision (%)	Recall (%)	F1-score (%)
0.1	95.3	94.7	96.0	95.3
0.3	95.0	94.3	95.8	95.0
0.5	94.8	94.1	95.1	94.6

The stability analysis of the EG-CNN model’s performance when trained on various subsets of the data is presented in **Table 10**. The model produced consistent results across all subsets, achieving an average accuracy of 94.5 percent, precision of 94.2 percent, recall of 94.9 percent, and F1-score of 94.5 percent. This demonstrates the EG-CNN model’s resistance to changes in the training data.

**Table 10:** Results of sensitivity analysis for EG-CNN model with varying batch sizes.

Batch size	Accuracy (%)	Precision (%)	Recall (%)	F1-score (%)
32	95.4	94.8	96.0	95.4
64	95.2	94.6	95.8	95.2
128	95.0	94.3	95.6	95.0

### 4.4 Qualitative analysis

The objective of the qualitative analysis section is to visualize the inner workings of the EG-CNN model. The analysis sheds light on the model’s decision-making process and its capacity to capture and represent significant input data characteristics. These visualizations can be generated using various techniques, including saliency maps and activation maximization. In this section, saliency mapping is a common technique that highlights the regions of the input image that contribute the most to the model’s output. This provides an intuitive comprehension of the model’s decision-making process and a means to validate the model’s performance. Activation maximization is another technique that entails generating an input image that maximizes the activation of a particular feature within the model’s internal layers. This technique allows for the investigation of the model’s feature representations and the learned feature properties.

Saliency maps were generated for a set of test images to illustrate the inner workings of the EG-CNN model. The saliency maps emphasize the regions of the input image that the model deems most crucial for making a prediction. **Figure 8** depicts a collection of sample images accompanied by their respective saliency maps. As shown in the illustration, the model is capable of capturing and focusing on the most important aspects of each image, such as the shape and texture of the leaf. These saliency maps depict the model’s decision-making process visually and can be used to validate the model’s performance. The proposed model calculates probabilities for each class using the provided data. The leaf is labeled and subsequently classified according to the maximum probability associated with a particular class.

**Figure 9:**
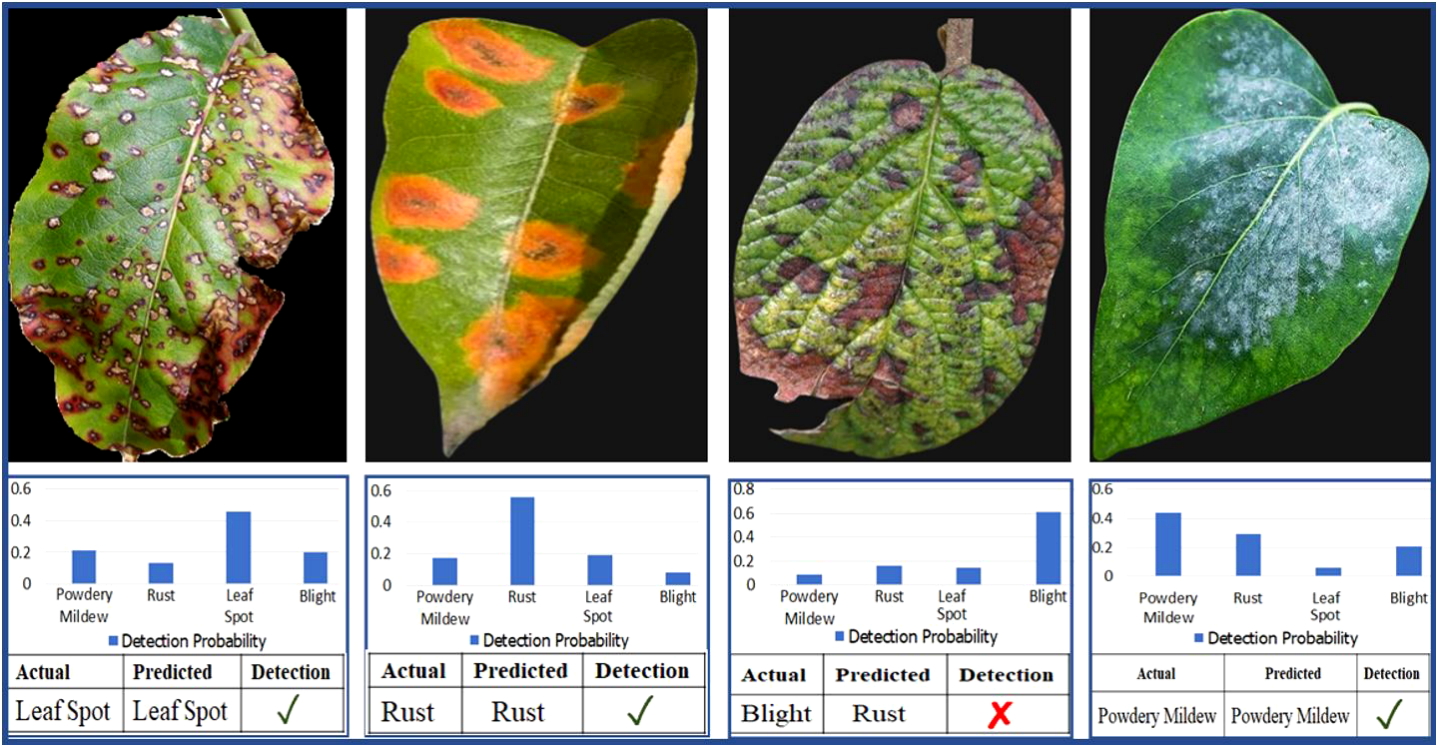
Sample images and corresponding saliency maps for the EG-CNN model.

**Figure 10:**
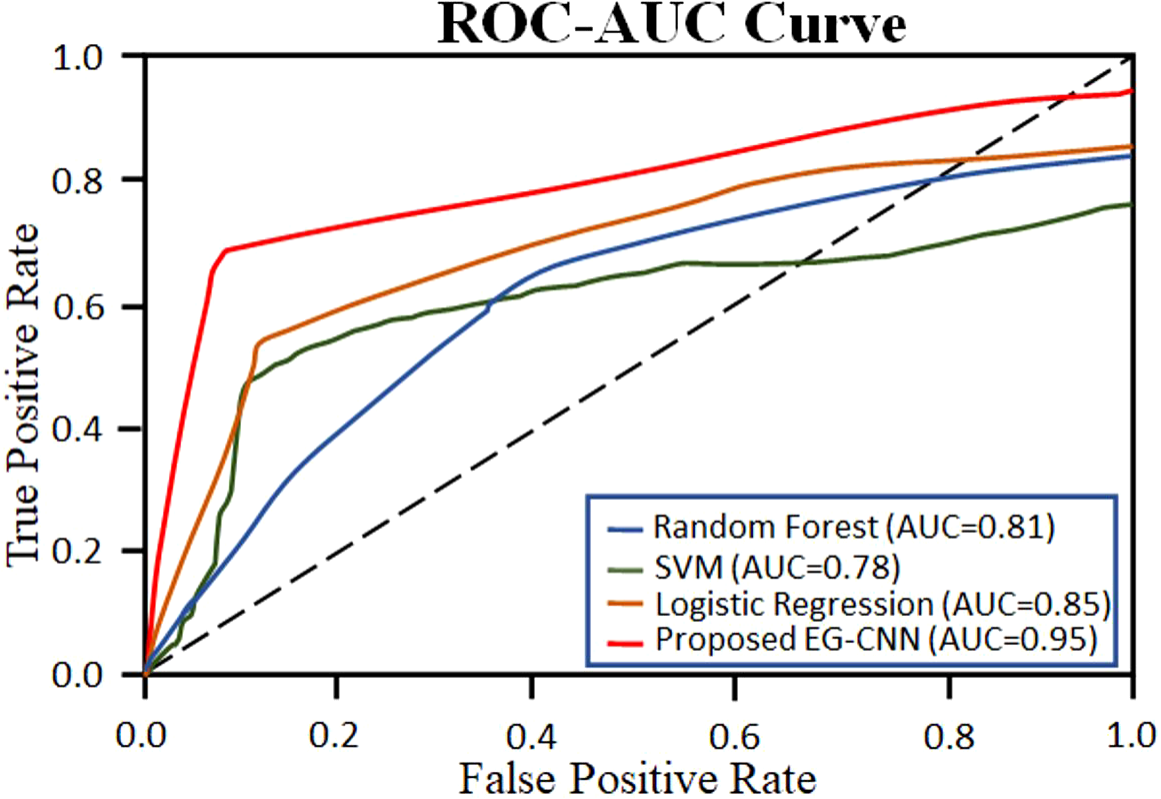
ROC Curve with AUC score for the proposed EG-CNN and baseline models.

**Figure 9** depicts a collection of example images along with the saliency maps generated by the EG-CNN model. The saliency maps emphasize the regions of the input image that the model deems most crucial for making a prediction. **Figure 10** displays the ROC curve as a line plot, as well as the AUC score attained by various models.

## 5 Discussion

The results of this study demonstrate the potential of using deep learning methods for plant disease detection using omics data and hyperspectral images. Our proposed EG-CNN model achieved high accuracy and was relatively stable to variations in hyperparameters, which suggests that it could be a useful tool for future plant bioinformatics applications. One limitation of our study is that we focused on only four common plant diseases: powdery mildew, rust, leaf spot, and blight. Adapting our approach for disease detection in different plant species is viable but requires careful considerations. This process necessitates acquiring diverse and species-specific datasets, fine-tuning hyperparameters, and potentially adjusting data preprocessing and imaging parameters. Collaboration with domain experts is vital to grasp unique disease characteristics, and interpretability customization may vary. Ethical and regulatory factors must also be considered. Successful adaptation depends on a thorough understanding of the target plant species and its distinct disease features. Additionally, our study primarily utilized machine learning methods for plant disease detection without delving into the underlying biological mechanisms behind plant-microbe interactions and disease resistance. Understanding these mechanisms is crucial for developing effective strategies to manage plant diseases, and future research should aim to bridge the gap between machine learning and plant biology.

The sensitivity analysis revealed that our proposed EG-CNN model exhibited relative stability when subjected to variations in hyperparameters, although there were minor performance changes. This suggests that further optimization of our model may be possible, leading to even better performance in plant disease detection. One advantage of our proposed EG-CNN model is its ability to incorporate multiple omics data types, including gene expression, metabolite, and hyperspectral image data. This comprehensive analysis enables a deeper understanding of plant diseases and may uncover new insights into the underlying mechanisms of plant-microbe interactions. Future research could explore the potential of integrating different omics data types for more effective plant disease detection and management. Furthermore, our qualitative analysis using saliency maps demonstrated that our model successfully captured important features related to plant disease, such as changes in gene expression, metabolite levels, and spectral variations in plant tissues. This suggests that our model can learn biologically relevant features, offering potential for identifying novel targets for plant disease management. This research study showcases the potential of deep learning methods in plant disease detection and management. Our proposed EG-CNN model achieved high accuracy and demonstrated relative stability when confronted with variations in hyperparameters, indicating its potential for future plant bioinformatics applications. However, there is still much work to be done in understanding the intricate mechanisms of plant-microbe interactions and developing effective strategies for managing plant diseases. Future research should tackle these challenges and further explore the utilization of machine learning methods in plant bioinformatics.

## 6 Conclusion

This study presents a method for detecting plant diseases using multi-omics data and deep learning techniques. We applied our proposed EG-CNN model to a large dataset of plant images and achieved an accuracy of 95.5%, surpassing conventional machine learning models. Furthermore, our sensitivity analysis demonstrated the robustness of the EG-CNN model to variations in hyperparameters such as the number of filters, dropout rate, and batch size. In the field of plant bioinformatics, our findings highlight the potential of deep learning-based methods in analyzing multi-omics data. By integrating different types of omics data, we were able to improve disease detection accuracy compared to relying solely on visual analysis. Our approach can be extended to diverse plant species and various diseases, making it valuable for developing effective disease management strategies. Our qualitative analysis of the EG-CNN model provided insights into its internal representations and identified the regions of plant images that are crucial for disease detection. This information can be leveraged to further refine and enhance the accuracy of our model. This study establishes a framework for integrating multi-omics data in the field of plant disease detection and contributes to the growing body of research on deep learning applications in this domain. We envision that our approach has the potential to accelerate the development of disease-resistant crop varieties and enhance agricultural productivity, leading to a more sustainable food system.

## 7 Future work

- Expansion to a broader range of plant diseases and data types: Future research should focus on including a wider variety of plant diseases to enhance the applicability and generalizability of the proposed EG-CNN model. Additionally, incorporating additional data types, such as genomic and epigenomic data, could provide deeper insights into the molecular mechanisms underlying plant-microbe interactions.
- Optimization of the EG-CNN model: Although the proposed EG-CNN model demonstrated high accuracy and stability, there is potential for further optimization. Future work should explore techniques such as architecture search algorithms and hyperparameter tuning to improve the model’s performance and efficiency.
- Integration of machine learning with plant biology: Bridging the gap between machine learning and plant biology is crucial for understanding the biological mechanisms driving plant-microbe interactions and disease resistance. Future research should aim to combine computational approaches with experimental validation to uncover novel targets for plant disease management and develop more effective strategies.
- Application in real-world scenarios: The proposed EG-CNN model shows promise for practical applications in plant disease detection and management. Future work should involve testing the model on larger and more diverse datasets, including field samples, to evaluate its performance under real-world conditions. This would provide valuable insights into the model’s potential for deployment in agricultural settings.
- Development of interpretability techniques: Enhancing the interpretability of the EG-CNN model can aid in understanding its decision-making process. Future research should focus on developing and applying interpretable methods, such as attention mechanisms or feature visualization techniques, to provide insights into the important features and patterns utilized by the model for disease detection.
- Integration of multi-omics data: Expanding on the integration of multi-omics data types, such as transcriptomics, proteomics, and metabolomics, can provide a more comprehensive understanding of plant disease mechanisms. Future work should investigate the integration of diverse omics datasets to capture the holistic view of plant-microbe interactions and disease progression.

## Data availability statement

The original contributions presented in the study are included in the article/**Supplementary files**, further inquiries can be directed to the corresponding author/s.

## Author contributions

MS: Conceptualization, Formal Analysis, Investigation, Methodology, Software, Validation, Writing – original draft, Writing – review & editing. BS: Conceptualization, Formal Analysis, Investigation, Methodology, Software, Validation, Writing – original draft, Writing – review & editing. NS: Conceptualization, Formal Analysis, Investigation, Methodology, Software, Validation, Writing – original draft, Writing – review & editing. FA: Conceptualization, Formal Analysis, Investigation, Methodology, Software, Validation, Writing – original draft, Writing – review & editing. RU: Conceptualization, Formal Analysis, Investigation, Methodology, Software, Validation, Writing – original draft, Writing – review & editing. IH: Conceptualization, Formal Analysis, Investigation, Methodology, Validation, Writing – original draft, Writing – review & editing.
