# Supplementary material for: Deep learning for plant bioinformatics: an explainable gradient-based approach for disease detection
Source: Front Plant Sci. 2023 Oct 13;14:1283235. doi: 10.3389/fpls.2023.1283235 (PMC10612337; doi:10.3389/fpls.2023.1283235)
Supplement: Supplementary file 1 [file Table_1.docx]

Table 1: Summary of omics data collected.

| **Omics data** | **Collection method** | **Number of features** |
| --- | --- | --- |
| Gene expression | RNA sequencing | 20,000 |
| Metabolites | GC-MS | 500 |
| Hyperspectral images | Spectrometer | 100 |
