# Supplementary material for: Deep learning for plant bioinformatics: an explainable gradient-based approach for disease detection
Source: Front Plant Sci. 2023 Oct 13;14:1283235. doi: 10.3389/fpls.2023.1283235 (PMC10612337; doi:10.3389/fpls.2023.1283235)
Supplement: Supplementary file 2 [file Table_2.docx]

Table 2: Top contributing gene expression features ranked by their gradient values.

| **Rank** | **Gene Expression Feature** | **Gradient Value** |
| --- | --- | --- |
| 1 | AT4G34270 | 0.75 |
| 2 | AT3G05720 | 0.67 |
| 3 | AT5G48480 | 0.58 |
| 4 | AT2G21260 | 0.52 |
| 5 | AT1G53230 | 0.49 |
| 6 | AT5G47910 | 0.47 |
| 7 | AT5G47390 | 0.44 |
| 8 | AT5G50915 | 0.42 |
| 9 | AT3G51860 | 0.39 |
| 10 | AT3G01540 | 0.37 |
