# Supplementary material for: Deep learning for plant bioinformatics: an explainable gradient-based approach for disease detection
Source: Front Plant Sci. 2023 Oct 13;14:1283235. doi: 10.3389/fpls.2023.1283235 (PMC10612337; doi:10.3389/fpls.2023.1283235)
Supplement: Supplementary file 3 [file Table_3.docx]

Table 3: Hardware and Software Configuration.

| **Component** | **Specification** |
| --- | --- |
| CPU | Intel Core i7-10700K |
| GPU | NVIDIA GeForce RTX 3080 |
| RAM | 32 GB DDR4 |
| Operating System | Windows 10 Pro |
| Framework | TensorFlow 2.5 |
| Libraries | NumPy, Pandas, Matplotlib |
